# Ted Janssen and aperiodic crystals

**DOI:** 10.1107/S2053273318016765

**Published:** 2019-02-06

**Authors:** Marc de Boissieu

**Affiliations:** a Université Grenoble Alpes, CNRS, SIMaP, 1130 Rue de la Piscine, BP 75, 38402 St Martin d Heres, France

**Keywords:** aperiodic crystals, superspace crystallography, lattice dynamics, phasons, Ted Janssen

## Abstract

This article reviews some of Ted Janssen’s (1936–2017) major contributions to the field of aperiodic crystals. Aperiodic crystals are long-range ordered structures without 3D lattice translations and encompass incommensurately modulated phases, incommensurate composites and quasicrystals. Together with Pim de Wolff and Aloysio Janner, Ted Janssen invented the very elegant theory of superspace crystallography that, by adding a supplementary dimension to the usual 3D space, allows for a deeper understanding of the atomic structure of aperiodic crystals. He also made important contributions to the understanding of the stability and dynamics of aperiodic crystals, exploring their fascinating physical properties. He constantly interacted and collaborated with experimentalists, always ready to share and explain his detailed understanding of aperiodic crystals.

## Abstract 2

Ted Janssen’s contributions to the field of aperiodic crystals are reviewed.

## 1. Introduction   

Ted Janssen, 81 years old, passed away on 29 September 2017 of a rapid leukaemia (Souvignier, 2018 ▸). With him, crystallography lost the last of the founding fathers of *n*-dimensional crystallography (or superspace crystallography) after Pim de Wolff (Janssen & Tuinstra, 1998 ▸) and, most recently, Aloysio Janner (Janssen, 2016 ▸). This article reviews some of the major contributions Ted Janssen made to the field his scientific life was fully dedicated to: aperiodic crystals.

Aperiodic crystals are long-range ordered structures which lack 3D periodicity. Although there are still on-going discussions and research on precisely how to define and characterize a long-range ordered structure, we will use in the following the definition of a crystal given by the IUCr Commission on Aperiodic Crystals in their report for the year 1991 (International Union of Crystallography, 1992 ▸; see page 928): ‘by crystal we mean any solid having an essentially discrete diffraction diagram, and by aperiodic crystal we mean any crystal in which 3D periodicity can be considered to be absent’. In other words, an aperiodic crystal is characterized by a diffraction pattern consisting mainly of sharp Bragg peaks and requiring more than three integer indices to be indexed properly.

**Figure 1:**
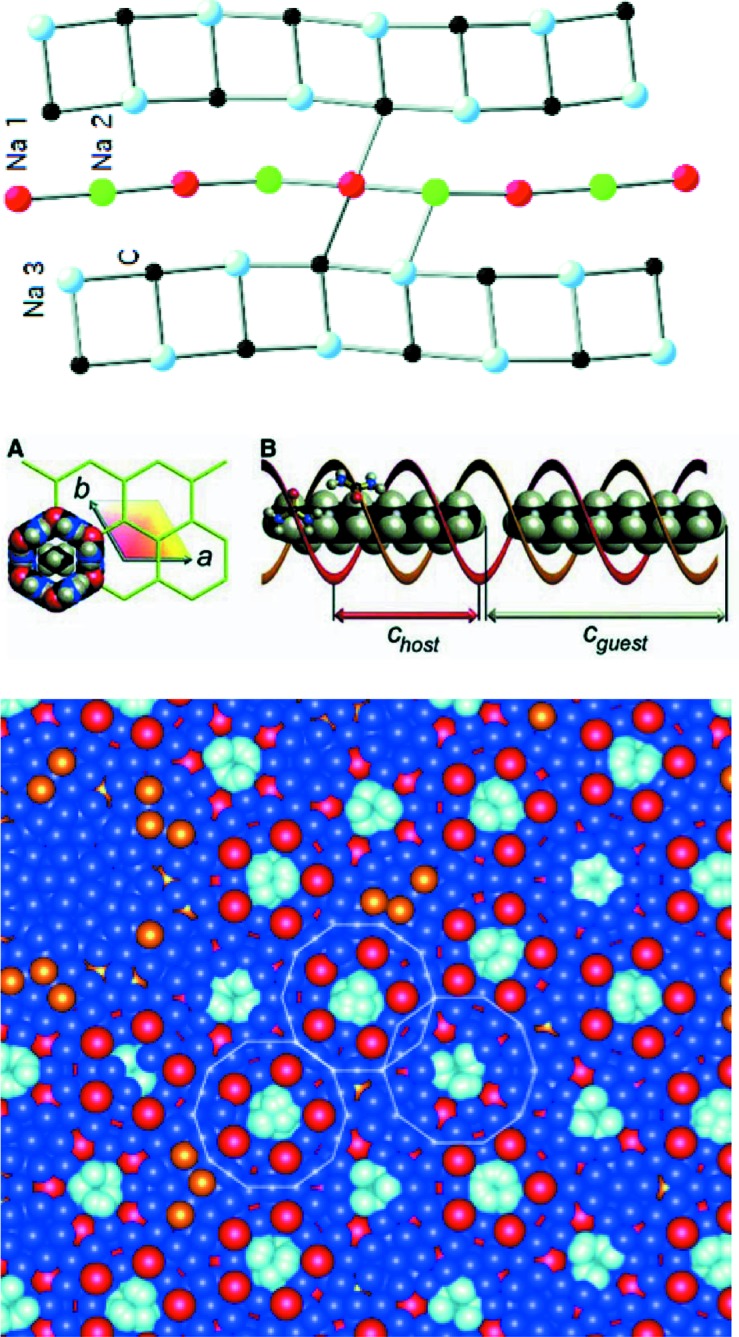
Illustration of the atomic structure of the three categories of aperiodic crystals as seen in the physical space. A more compact and detailed description is achieved in the superspace description. From top: incommensurately modulated structure found in Na_2_CO_3_ [courtesy of Gervais Chapuis from Dusek *et al.* (2003 ▸)], composite with host and guest channels in the alcane–urea compound [courtesy of B. Toudic, from Toudic *et al.* (2008 ▸), reprinted with permission from AAAS], atomic plane of the icosahedral Zn—Sc quasicrystal [courtesy of T. Yamada, from Yamada *et al.* (2016 ▸)].

Aperiodic crystals are usually classified in three different categories: incommensurately modulated structures, incommensurate composite structures and quasicrystals (see Fig. 1 ▸ for an illustration). In an incommensurately modulated structure a periodic structure is subject to one or several modulations whose period is irrational with the basic periodicity. It can be a position or a composition modulation, with a modulation function whose shape can be in the simplest case a sinusoidal function, but is in general much more complex. An incommensurate composite crystal consists of two or more subsystems, each being incommensurately modulated because of the interaction between the two subsystems. Simple cases are host–guest structures. Finally quasicrystals, discovered by Shechtman *et al.* (1984 ▸), constitute the third class of aperiodic crystals. Quasicrystals are generally described as aperiodic crystals with a symmetry incompatible with lattice periodicity, although this is in principle not necessary (Janssen *et al.*, 2007 ▸, 2018 ▸): in particular, icosahedral, decagonal, dodecagonal and octahedral symmetry have been observed experimentally.

Long ago considered as rare and ‘strange’ structures, aperiodic crystals are nowadays observed everywhere in many different systems. The advances of 2D detectors for X-ray and neutron diffraction as well as progress in electron diffraction have been certainly key for this, but the possibility of solving the structure using the superspace theory developed by A. Janner, T. Jannsen and P. de Wolff, which was then implemented in software, has also been crucial in that perspective. It is not possible to give an exhaustive list of the hundreds of aperiodic crystal structures discovered and solved, but aperiodic crystals are found, for instance, in single elements under pressure, minerals, oxides, intermetallics, organic compounds, soft condensed matter and in many different functional systems such as ferroelectrics, multi-ferroics, high-*T*_c_ superconductors, charge-density waves, magnetically ordered systems and even protein crystals [see also the already long list published in Janner & Janssen (1977 ▸) more than 40 years ago and the *Acta Cryst.* B web page dedicated to incommensurately modulated structures, http://publcif.iucr.org/cifmoldb/mscif/]. The influence of the aperiodic order on physical and chemical properties is still under debate, but aperiodic crystals cannot be ignored any longer, nor can they be considered as ‘bad crystals’!

## 2. A brief history of aperiodic crystals   

Soon after the discovery of X-ray diffraction by von Laue and its use for structure determination by the Braggs (son and father), detailed investigations of the observed diffraction photographs pointed to broad and diffuse spots or streaks, first observed by Friedrich *et al.* (1913 ▸), which could not be interpreted as regular Bragg peaks. Following an interpretation proposed by Faxen in 1923 ▸, these streaks and diffuse spots were shown to be the result of thermal vibrations and phonons by two independent studies (Laval, 1939 ▸; Preston, 1939 ▸); this is the well known thermal diffuse scattering. A detailed historical description of this discovery can be found in the article by Lonsdale (1942 ▸).

**Figure 2:**
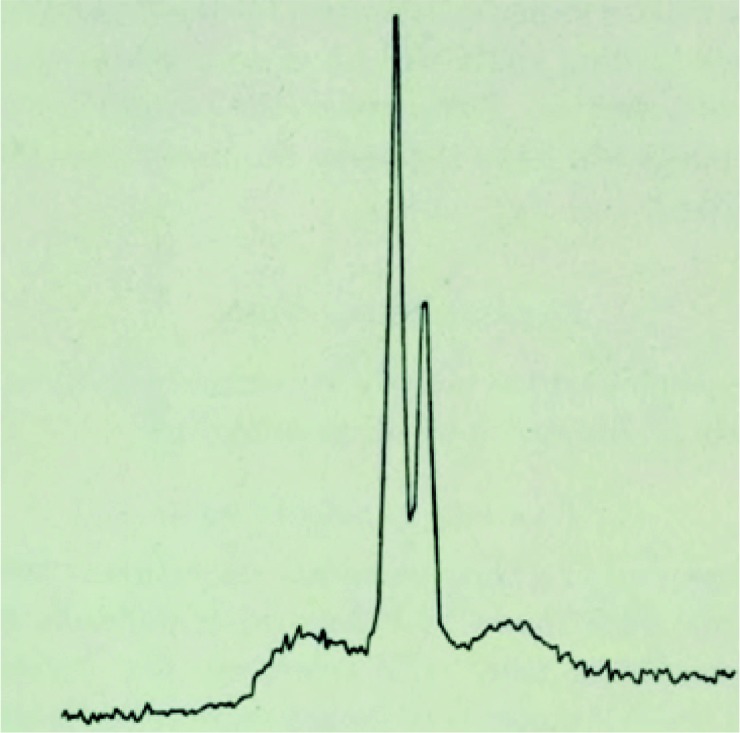
Illustration of one of the first modulated phases observed in a metallic alloy by Dehlinger [from Daniel & Lipson (1943 ▸)]. The two satellite reflections, a signature of the modulated state, are clear.

Meanwhile in 1927 U. Dehlinger, working on cold-worked metallic alloys at the University of Stuttgart, observed side bands in a Debye–Scherrer diffraction pattern. The correct interpretation of this observation was given in the same article as resulting from a sinusoidal distortion of the periodic lattice and may be considered to be the first observation of a modulated structure; it was most likely incommensurate, although this is not discussed in the article (Dehlinger, 1927 ▸). A more detailed theoretical analysis of the effect of a modulation of a periodic structure was given by Kochendörfer (1939 ▸). A detailed description of the modulated state, either displacive or compositional, and its qualitative and quantitative comparison with experimental data obtained for heat-treated Cu–Fe–Ni is given by Daniel & Lipson (1943 ▸), as illustrated in Fig. 2 ▸. Periodic structure subject to a periodic modulation was thus recognized and understood rather early on, and presented in detail, for instance, in the chapter *Diffraction by lattices with periodic distortions* in the book *Optical Principles of the Diffraction of X-Rays* by R. W. James, first published in 1948 (James, 1962 ▸); a detailed computation of the satellite intensity is also found in Korekawa (1967 ▸). That the period of the modulation can be incommensurate with respect to the underlying periodic lattice was then best demonstrated looking at the temperature variation of the modulated wavevector in helical magnetic systems [MnAu_2_ (Herpin *et al.*, 1960 ▸), Er (Will, 1968 ▸)], and in NaNO_2_ and Na_2_CO_3_ crystals. In particular, in Na_2_CO_3_ the incommensurability of the modulation wavevector with the underlying lattice was clearly demonstrated (Brouns *et al.*, 1964 ▸). At the start of the 1970s, several incommensurately modulated phases were discovered and studied in quite different systems: *A*_2_ *B* *X*_4_ (Rb_2_WO_4_, Rb_2_ZnCl_4_, Rb_2_ZnBr_4_), NaNO_2_, thio­urea, plagioclase, K_2_Pt(CN)_4_BrO_3_, TaS_2_, TaTe_2_, NbSe_2_, Cr, MnAu_2_, Er, Tm *etc.* [see Table 1 and references therein in Janner & Janssen (1977 ▸)]. However, it was the articles by de Wolff (1977 ▸, 1974 ▸) and Janner & Janssen (1977 ▸) that really highlighted the incommensurability and its relation with symmetry.

Incommensurate composite structures with two or more interacting subsystems were first identified in fool’s gold (Hg_3−δ_AsF_6_), where mercury 1D chains ordered below 120 K (Hastings *et al.*, 1977 ▸; Pouget *et al.*, 1978 ▸), and in (TTF)_7_I_5_ (Johnson & Watson, 1976 ▸), where I ions form guest chains along the *c* axis of the TTF host lattice.

The third class of aperiodic crystals, quasicrystals, received much attention with the publication of the discovery of an icosahedral quasicrystal by Shechtman *et al.* (1984 ▸). Observed first only for rapidly solidified alloys, they were then obtained as equilibrium phases and large single grains in different intermetallic alloy systems and also in soft condensed matter (Janssen *et al.*, 2007 ▸, 2018 ▸).

As stated in the introduction, aperiodic crystals are nowadays observed everywhere in many different systems.

## 3. The birth of superspace crystallography and its development.   

**Figure 3:**
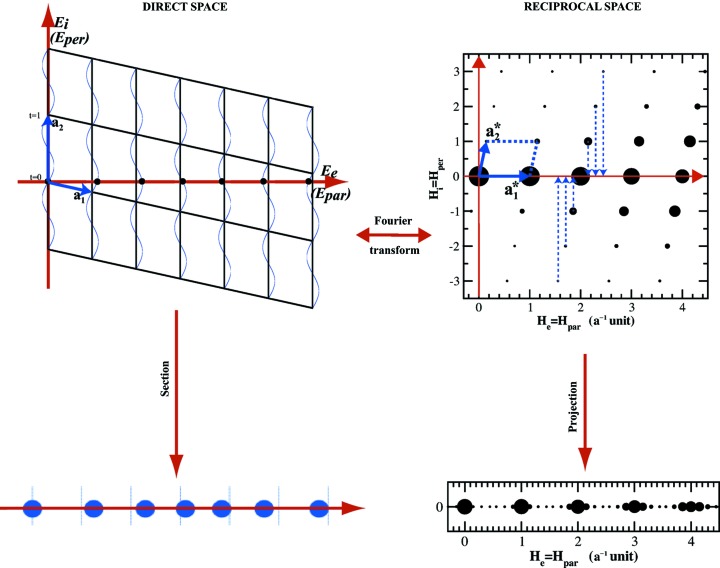
Illustration of superspace crystallography in the case of a 1D incommensurately modulated phase. Bottom left: the modulated phase where atoms are displaced with respect to the ideal periodic positions, shown as vertical lines. Bottom right: diffraction pattern of the modulated phase. Satellite reflections are visible around each Bragg peak located at the positions in reciprocal space of the undeformed periodic structure. The upper part shows the 2D embedding of the modulated chain (left panel) and of the diffraction pattern (right panel). The wavy lines are called atomic surfaces and represent the modulation. The 1D incommensurately modulated structure is obtained as a section of the 2D one. On the other hand, the 1D diffraction pattern is obtained as a projection of the 2D one.

A very good account of the history and birth of superspace crystallography can be found in the review article by Janssen & Janner (2014 ▸). In the late 1960s and early 1970s, A. Janner and T. Janssen worked theoretically, in Nijmegen, on the problem of crystallographic groups in space and time and thus in 4D. At the same time, P. M. de Wolff, in Delft, studying the incommensurate phase Na_2_CO_3_, came to the idea of describing this phase in a 4D space. At the IUCr Congress held in 1972 in Kyoto they both presented their results and realized they were talking about the same theory. The superspace theory of aperiodic crystals was born with its two essential ‘ingredients’: (i) a description in a space with dimension larger than three, named superspace, where the structure is described as a decorated periodic structure; and (ii) the derivation of the symmetry of the aperiodic phase in the superspace and its superspace group leading to specific constraints for the modulation polarization, for instance, and to possible extinctions in the diffraction pattern (de Wolff, 1977 ▸; Janner & Janssen, 1977 ▸, 1980*a* ▸,*b* ▸; de Wolff *et al.*, 1981 ▸). An illustration of the procedure and concept, with a simple example of the 1D displacive modulated chain and its representation in a 2D periodic space, is given in Fig. 3 ▸.

Soon after the basis of the superspace theory was established for incommensurately modulated phases, Janner and Janssen extended it to the case of incommensurately modulated composite structures (Janner & Janssen, 1980*a* ▸,*b* ▸) while a first list of all superspace groups in 3 + 1 dimensions was published by de Wolff *et al.* (1981 ▸). It is important to note that this is different from the list of all space groups in four dimensions, which consist of 4783 space groups; the crystallographic ones amount to 756. It is also important to stress the difficulty, from the mathematical point of view, in properly deriving the entire set of space groups, a task that benefitted greatly from the PhD work of Ted Janssen on crystallographic groups in space and time published in a series of three articles (Janssen *et al.*, 1969*a* ▸,*b* ▸; Janssen, 1969 ▸).

This discovery nicely illustrates how a theoretical and mathematical research topic became one of the most beautiful recently developed crystallographic theories thanks to cooperation between the experimentalist and crystallographer Pim de Wolff and the two theoreticians Ted Janssen and Aloysio Janner. This also illustrates what has been a constant in Ted Janssen and Aloysio Janner’s way of conducting research: they were always ready to share their knowledge and interact with experimentalists to interpret data. The importance of superspace crystallography theory was recognized by the Aminoff Prize, which was awarded to the three of them in 1998, and the prestigious Ewald Prize, awarded to Ted Janssen and Aloysio Janner at the Montreal IUCr Congress in 2014.

**Figure 4:**
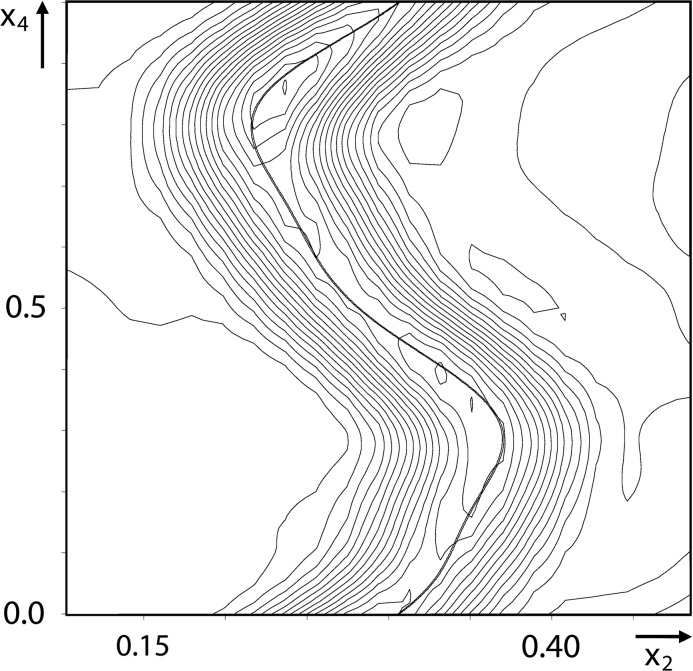
Example of part of the superspace description of the atomic structure of Fig. 1 ▸ for Na_2_CO_3_ (Dusek *et al.*, 2003 ▸) as shown in a section of the 4D space.

The field of incommensurately modulated phases and incommensurate composites took advantage of the newly developed theory, in particular thanks to its implementation in software dedicated to the structure determination of incommensurately modulated structures, followed by incommensurate composite structures [*Remos82* (Yamamoto, 1982 ▸); *Jana* (Petříček *et al.*, 2014 ▸)]. However, it took some time to fully develop and this was ready to use by the end of the 1980s, when quasicrystals had already been discovered [see the nice review by A. Yamamoto, who also contributed much to the field of aperiodic crystals (Yamamoto, 1996 ▸)]. The compact description of a periodic space of incommensurately modulated and incommensurate composite crystals has also allowed a detailed understanding of the crystal chemistry at play in these compounds. Indeed, variation of interatomic distances and of local environments are easily deduced from high-dimensional models [see the books by van Smaalen (2012 ▸) and Janssen *et al.* (2007 ▸, 2018 ▸)]. An example of a description of the Na_2_CO_3_ structure is displayed in Fig. 4 ▸.

The publication of the discovery of an icosahedral quasicrystal by Shechtman *et al.* (1984 ▸) was also a landmark. Indeed in this case, unlike for modulated or composite incommensurate phases, no underlying periodic lattice can be defined. Almost immediately after the publication, interpretation of the icosahedral quasicrystal diffraction pattern in terms of Penrose tiling (Levine & Steinhardt, 1984 ▸; Kramer & Neri, 1984 ▸) and also in terms of superspace crystallography in six dimensions was proposed (Bak, 1985*a* ▸,*b* ▸; Kalugin *et al.*, 1985*a* ▸,*b* ▸; Duneau & Katz, 1985 ▸; Elser, 1985 ▸). Although Bak had pointed out very early on the similarity of the approach with the one for incommensurately modulated structures, it took some time before the link could be made, and in some sense it could be said that superspace crystallography was rediscovered for quasicrystals. Ted Janssen, in a very important article, did show that quasicrystals are indeed of the same ‘family’ as incommensurately modulated structures and that the superspace theory is the same for all aperiodic crystals (Janssen, 1986*a* ▸,*b* ▸). The shape of the atomic surface (or occupation domains) is different though: while the structure is described by 1D atomic surfaces for incommensurately modulated structures, it turns out to be a 2D or 3D object in the case of quasicrystals. Ted Janssen also wrote one of the first complete and beautiful reviews on quasicrystals, *Aperiodic crystals: a contradictio in terminis?*, presenting in a very detailed and complete manner superspace crystallography, its application for structure determination, and also other topics such as quasicrystal crystal shapes and their indexing and physical properties (Janssen, 1988 ▸). This article certainly constitutes one of the landmarks in the field.

Ted Janssen also explored the frontiers of the domain, in particular the occurrence of fractal atomic surface boundaries in some self-similar structures (Luck *et al.*, 1993 ▸).

### 3.1. Ted Janssen’s contribution to the crystallographic community   

Ted Janssen also had a very important role in providing tools and nomenclature for use by crystallographers. He was, in particular, responsible for the Subcommittee on the Nomenclature of *n*-Dimensional Crystallography (Janssen *et al.*, 1999 ▸; Janssen, Birman *et al.*, 2002 ▸). This is very important work, allowing a standardized publication of *n*-dimensional superspace crystallography that can be shared among scientists.

He also contributed to three very important chapters in *International Tables for Crystallography*. Together with A. Janner, A. Looijenga-Vos and P. M. de Wolff, he wrote a detailed chapter introducing the field of incommensurately modulated structures (Janssen *et al.*, 2006 ▸).

Ted Janssen also wrote two pedagogical sections introducing group representations (Janssen, 2013*a* ▸) and the tensor properties in quasiperiodic structures (Janssen, 2013*b* ▸).

## 4. Physical properties and phase transitions of aperiodic crystals   

What is the influence of the long-range aperiodic order on physical properties? Are there new and characteristic signatures of the long-range aperiodic order on physical properties? In particular, what are the excitation spectrum and the dynamics of an aperiodic crystal? What are the driving forces stabilizing the aperiodic long-range order? Can aperiodic long-range order propagate with only finite-range forces?

All these questions were raised once it was realized that a modulation can be incommensurate. They have been much debated in the field of quasicrystals, but the questions and problems raised are the same for all aperiodic crystals. From the very beginning, after the development of superspace crystallography, Ted Janssen tackled these questions and explored many of the facets of these problems for the different categories of aperiodic crystals, always proposing elegant theoretical answers. This is a field where he also collaborated with several groups of experimentalists, always keen to confront his theoretical results with experiment.

### 4.1. Excitation spectra, phonons and phasons in aperiodic crystals   

Solid-state physics and the derivation of physical properties rely on the Bloch-wave extension and thus on the periodicity. These tools cannot be used for the study of aperiodic crystals. Attempts were made to use the superspace approach to tackle these problems, but most of the derivation has been carried out on periodic ‘approximants’ of aperiodic crystals, studying the evolution of the excitation spectrum as the cell size increases.

**Figure 5:**
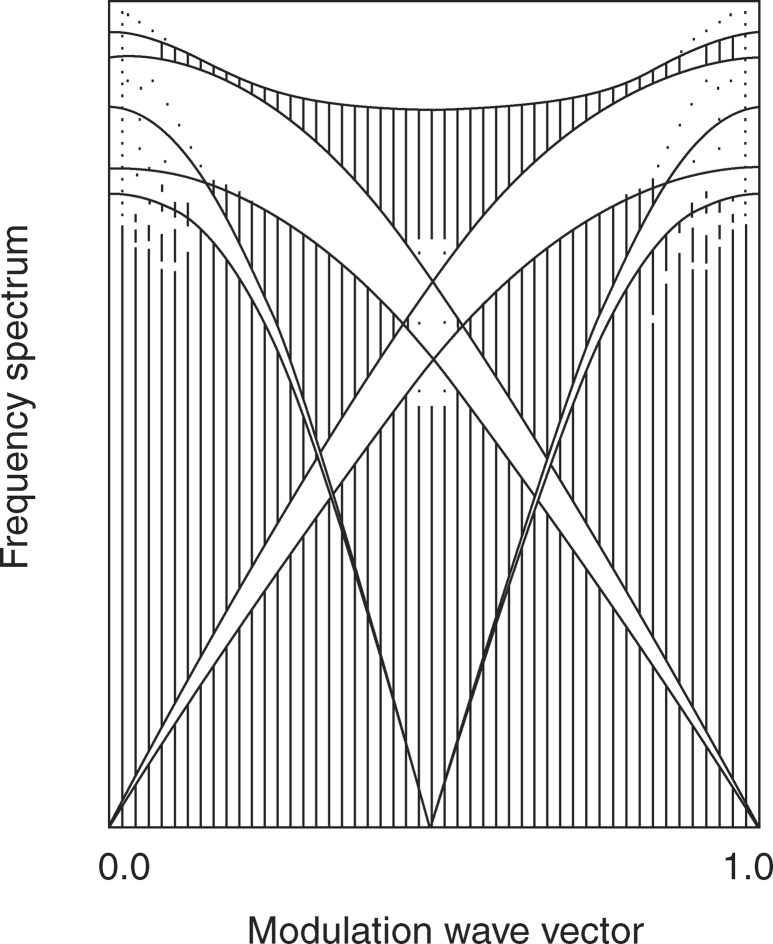
Excitation spectrum of the modulated 1D chain as a function of the modulation vector. For each value of the modulation wavevector the spectrum is projected on a vertical energy axis. Large-gap openings are observed from de Lange & Janssen (1981 ▸).

With hand-waving arguments on a 1D chain, starting from a single atom periodic chain, let us consider a modulation. If the modulation period is rational and just half the period of the periodic chain, then the initial cell is doubled (this is a rational approximant) and a gap opens in the phonon dispersion relation. If the modulation is one third, two gaps will open and so on. In the limit of the irrational modulation and infinite cell, one thus expects an infinite number of gaps (de Lange & Janssen, 1981 ▸). The analysis carried out on these 1D modulated chains shows the fractal character of the excitation spectrum in the limiting aperiodic case on one hand, but also the rapidly vanishing size of the gaps on the other hand (de Lange & Janssen, 1981 ▸). This is illustrated in Fig. 5 ▸, which represents a projection of the spectrum on the vertical energy axis as a function of the modulation wavevector. A similar result was obtained for the Frankel–Kontorova model describing adsorbed atoms on a periodic substrate, for instance. In the weak-coupling regime the situation is the same as for the modulated chain.

Phason modes were first recognized as new excitations, characteristic of the aperiodic long-range order, for incommensurately modulated phases. Phasons are in this case related to the phase of the modulation, which in the superspace description corresponds to a shift in the internal or perpendicular direction and to the free-energy degeneracy of the system with respect to this direction. Phason modes can be viewed as perturbations with a polarization in the internal superspace direction, and are characteristic of all aperiodic crystals. A detailed description of phasons is beyond the scope of this article and the reader is referred to reviews such as that by Currat & Janssen (1988 ▸) or the book by Janssen *et al.* (2007 ▸, 2018 ▸).

**Figure 6:**
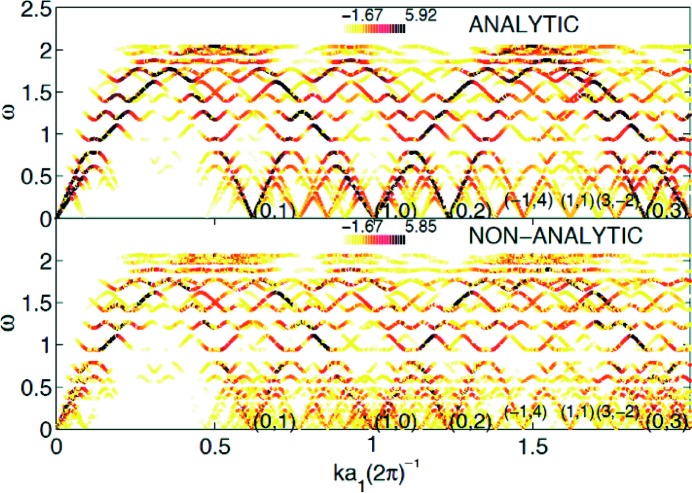
Simulation of the *S*(*Q*, *E*) response function in the double-chain model in the case of weak coupling (top) and strong coupling (bottom). Reprinted by permission from Springer Nature from Radulescu *et al.* (2002 ▸).

In the case of 1D modulated phases, the phason branch and its relation to a phonon-like vibration were indeed observed in a numerical simulation with short-range interaction stabilizing the incommensurate structure (Janssen & Tjon, 1982 ▸, 1983 ▸). The effect of the shape of the modulation was also shown in this model, with the occurrence of a phason gap for non-sinusoidal modulation. The second 1D system of interest is the Frankel–Kontorova model for which the phason branch only goes to zero in the regime of weak coupling (de Lange & Janssen, 1981 ▸). This model can also be viewed as a model of a composite with two different sublattices, but Ted Janssen and his co-worker, with the double-chain model, developed a much better composite approximation. This model allows for varying of the stiffness of the two sublattices together with their interaction strength. It turns out that the composite crystal is probably the most difficult case, as illustrated in Fig. 6 ▸. The elastic constants of and interaction between the two sublattices make it difficult to predict where the phason branch should be observed. However, a very detailed simulation, in particular taking into account the experimental setup, was carried out and discussed in term of sliding modes (Radulescu & Janssen, 1999 ▸; Radulescu *et al.*, 2002 ▸; Janssen, Radulescu & Rubtsov, 2002 ▸). Based on these simulations and on experimental results an indirect signature of the phason sliding mode could be shown in the alkane–urea incommensurate composite compound by Toudic, Lefort *et al.* (2011 ▸).

Ted Janssen has also pioneered simulations of the lattice dynamics in 3D models of icosahedral quasicrystals (Los & Janssen, 1990 ▸; Los *et al.*, 1993*a* ▸,*b* ▸). Simulations were performed on larger and larger periodic approximants showing a characteristic self-similar structure at low energy that resembled results obtained for the 1D Fibonacci chain. Simulations of the *S*(*Q*, *E*) function, measured by inelastic neutron scattering, were also carried out for comparison with experimental results. Again this illustrates the important role that Ted Janssen’s work has played for experimentalists, as illustrated in the review article by Quilichini & Janssen (1997 ▸).

### 4.2. Phase transitions and stability of aperiodic crystals   

**Figure 7:**
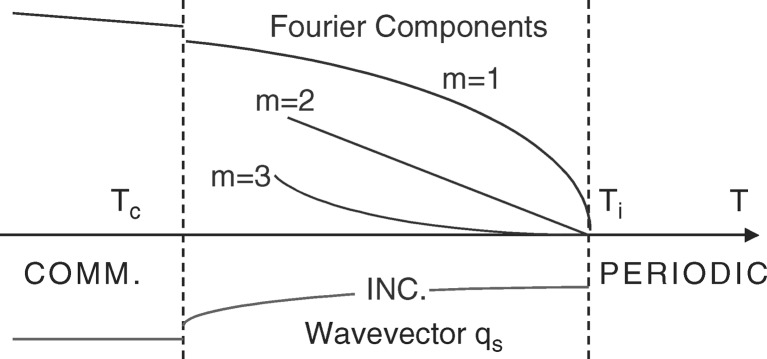
Typical temperature dependence and phase transitions leading to an incommensurately modulated phase. Starting from the high-temperature periodic phase, the incommensurately modulated phase forms below *T*_i_. As the temperature is decreased, the wavevector modulus changes. At the same time the modulation becomes more anharmonic, leading to higher Fourier components. At *T*_c_ the phase ‘locks in’ into a periodic approximant, the modulation wavevector being a rational value of the high-temperature periodic phase lattice constant.

Many incommensurately modulated phases are stable in a restricted temperature range. Generally, starting from a high-symmetry high-temperature phase, a modulated phase appears below a first transition *T*_i_ down to the temperature *T*_c_. Between *T*_i_ and *T*_c_ the wavevector of the modulated phase continuously varies, a proof that it is incommensurate, to lock on to a rational approximant below *T*_c_. At the same time the modulation function goes from a rather sinusoidal form to a more anharmonic form. Moreover, the formation of the incommensurately modulated phase is also frequently driven by a soft phonon mode showing up at high temperature that condenses at the modulation wavevector at *T*_i_ (see Fig. 7 ▸).

**Figure 8:**
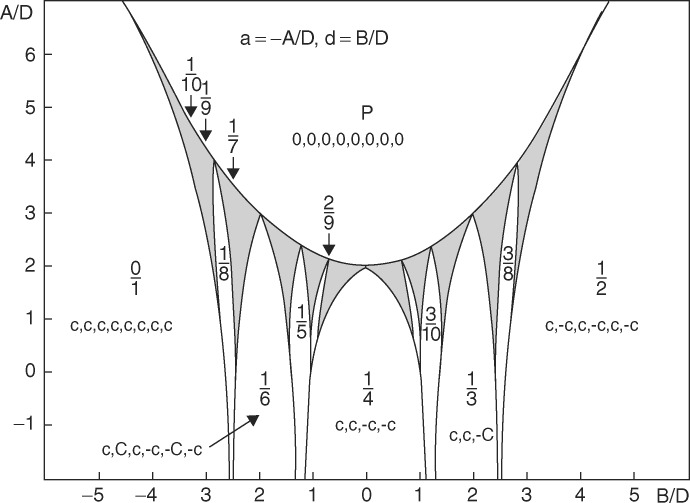
Phase diagram obtained with the DIFFOUR model. The grey areas correspond to the incommensurately modulated phase domain whereas other regions are locked-in periodic phases. Reprinted with permission from Janssen & Tjon (1982 ▸). Copyright (1982) by the American Physical Society. https://doi.org/10.1103/PhysRevB.25.3767.

While phenomenological Landau theory proved to be very efficient in explaining these transitions, the microscopic driving force leading to an incommensurate phase remained a mystery for a long time. A first model is the axial next-nearest neighbour Ising model (ANNNI), mostly derived for incommensurate magnetic structures, without any lattice dynamics. The discrete frustrated Φ^4^ model, abbreviated DIFFOUR, allows one to grasp the entire physics of these phase transitions. The model contains both a continuous part with a fourth-order polynomial and a first- and second-neighbour interaction in a plane. Without going into the details of the Hamiltonian, one of the important parts is the frustration term that might stabilize the incommensurate phases. The stable phases depend only on two parameters that can be mapped out in temperature and composition, for instance (Janssen & Tjon, 1981 ▸, 1982 ▸, 1983 ▸). By varying these two parameters and within a mean-field description of the model, a complete phase diagram is obtained as illustrated in Fig. 8 ▸. Since the dynamics are included in this model, it can be calculated: as expected a soft phonon mode builds up from the high-temperature phase and condenses at *T*_i_. Below *T*_i_ the dispersion relation displays the phason and amplitudon mode.

This model thus reproduces and provides an explanation for what is observed experimentally in ionic crystals or some ferroelectric compounds such as Na_2_CO_3_, K_2_SeO_4_ or Rb_2_ZnCl_4_. Not only is the phase transition reproduced but also the evolution of the structure as one goes from *T*_i_ to *T*_c_; changes in the modulation function and a chaotic state of discommensurations near *T*_c_ are fully accounted for. This beautiful description of phase transitions and stabilization mechanisms in aperiodic crystals is certainly one of the greatest achievements in the field.

Recently, Ted Janssen was much involved in interpreting the complex phase transitions observed in alkane–urea compounds (Toudic *et al.*, 2008 ▸). Indeed, in this case it was shown that a transition from an incommensurate phase to another incommensurate phase with one supplementary wavevector could be interpreted as a transition in the superspace, increasing its dimension and thus implying the internal (or phason) space degrees of freedom (Toudic, Rabiller *et al.*, 2011 ▸). In some sense this is a generalization of the Landau-type phase transition to the case of superspace crystallography, a case that has also been explored for quasicrystals.

Indeed, several complex phase transitions have been observed in quasicrystals, and Ted Janssen also gave a theoretical interpretation for these (Janssen, 1991 ▸).

## 5. Conclusion   

The study of aperiodic crystals is expanding in many directions. Aperiodic crystals are found everywhere in almost all systems, and the superspace crystallography approach is the way to understand their atomic structure and their crystal chemistry. The boundaries sometimes made between the different classes of aperiodic crystals, namely incommensurately modulated structures, incommensurate composite crystals and quasicrystals, are rather artificial and the entire field should really be considered as a unique one, as was constantly promoted by Ted Janssen. Indeed, large-amplitude incommensurate modulations, multiple *q* modulations, anharmonic ones, incommensurate composites with large intermodulations and quasicrystals certainly share similar structure and raise similar questions and problems.

The understanding of the physical properties of aperiodic crystals is still in its infancy, and here an approach that envisions all the aperiodic crystal classes should be promoted. In particular, the study of the dynamics and phason modes of aperiodic crystals, unique to this class of material, would certainly benefit from this joint approach. Finally, the driving forces that favour the long-range aperiodic order and the mechanisms allowing the aperiodic crystal growth are highly intriguing areas of study.

I had the chance to meet with Ted Janssen when I was a young researcher. He was always easy to approach, very kind in his answers, and keen to share his knowledge with young researchers and experimentalists. His scientific career was an illustration of the importance of a multi-disciplinary approach in crystallography and solid-state physics going from mathematics to experiments. Most of his scientific ideas are presented in the second edition of the book *Aperiodic Crystals* (Janssen *et al.*, 2018 ▸) that was finished just before he passed away. It was a great honour to work with Ted and Gervais Chapuis on this book, which owes a lot to Ted’s elegant approach. His work will certainly be an example for young theoretical researchers in crystallography.
